# Integrated bioinformatics analysis of nucleotide metabolism based molecular subtyping and biomarkers in lung adenocarcinoma

**DOI:** 10.3389/fimmu.2024.1430171

**Published:** 2024-08-01

**Authors:** Dayuan Luo, Haohui Wang, Zhen Zeng, Jiajing Chen, Haiqin Wang

**Affiliations:** ^1^ Department of Thoracic Surgery, The Second Xiangya Hospital of Central South University, Changsha, Hunan, China; ^2^ Hunan Key Laboratory of Early Diagnosis and Precise Treatment of Lung Cancer, The Second Xiangya Hospital of Central South University, Changsha, Hunan, China; ^3^ Department of Geriatrics, The Second Xiangya Hospital of Central South University, Changsha, Hunan, China; ^4^ Hunan Clinical Medical Research Center for Geriatric Syndrome, The Second Xiangya Hospital of Central South University, Changsha, Hunan, China

**Keywords:** lung adenocarcinoma, nucleotide metabolism, tumor microenvironment, immune microenvironment, AUNIP

## Abstract

**Background:**

Lung adenocarcinoma (LUAD), a predominant subtype of non-small cell lung cancers, continues to challenge treatment outcomes due to its heterogeneity and complex tumor microenvironment (TME). Dysregulation in nucleotide metabolism has been identified as a significant factor in tumorigenesis, suggesting its potential as a therapeutic target.

**Methods:**

This study analyzed LUAD samples from The Cancer Genome Atlas (TCGA) using Non-negative Matrix Factorization (NMF) clustering, Weighted Correlation Network Analysis (WGCNA), and various machine learning techniques. We investigated the role of nucleotide metabolism in relation to clinical features and immune microenvironment through large-scale data analysis and single-cell sequencing. Using *in vivo* and *in vitro* experiments such as RT-qPCR, Western Blot, immunohistochemistry, and subcutaneous tumor formation in mice, we further validated the functions of key nucleotide metabolism genes in cell lines and animals.

**Results:**

Nucleotide metabolism genes classified LUAD patients into two distinct subtypes with significant prognostic differences. The ‘C1’ subtype associated with active nucleotide metabolism pathways showed poorer prognosis and a more aggressive tumor phenotype. Furthermore, a nucleotide metabolism-related score (NMRS) calculated from the expression of 28 key genes effectively differentiated between patient outcomes and predicted associations with oncogenic pathways and immune responses. By integrating various immune infiltration algorithms, we delineated the associations between nucleotide metabolism signature genes and the tumor microenvironment, and characterized their distribution differences at the cellular level by analyzing single-cell sequencing dataset related to immunochemotherapy. Finally, we demonstrated the differential expression of the key nucleotide metabolism gene AUNIP acts as an oncogene to promote LUAD cell proliferation and is associated with tumor immune infiltration.

**Conclusion:**

The study underscores the pivotal role of nucleotide metabolism in LUAD progression and prognosis, highlighting the NMRS as a valuable biomarker for clinical outcomes and therapeutic responses. Specifically, AUNIP functions as a critical oncogene, offering a promising target for novel treatment strategies in LUAD.

## 1 Introduction

Lung cancer stands as one of the most prevalent forms of cancer worldwide, with lung adenocarcinoma (LUAD) being the predominant subtype among non-small cell lung cancers (1). Despite advances in therapeutic strategies in recent years, the long-term survival rates for patients with LUAD remain suboptimal. This challenge is partly due to the tumor heterogeneity and the complexity of the tumor microenvironment (TME), which collectively influence the biological behavior of tumors and response to treatments (2). Dysregulation of the cell cycle process is one of the fundamental mechanisms underlying tumorigenesis, making pathways closely associated with nucleotide metabolism viable targets for cancer therapy. Increasing evidence suggests that abnormalities in nucleotide metabolism coexist with other hallmarks of cancer, including metabolic reprogramming and immune evasion (3). Therefore, targeted therapy against key genes in nucleotide metabolism can not only inhibit the proliferation and progression of cancer cells but also reverse the aberrant metabolic state and restore immune surveillance. The rapid advancement of bioinformatics has provided new perspectives and tools for cancer research. Analyzing data from large-scale biomedical databases, researchers can unveil new characteristics of tumor biology, including alterations in tumor metabolism, the status of the immune microenvironment, and their correlations with patient prognosis (4).

This study utilizes LUAD samples from The Cancer Genome Atlas (TCGA) database, employing Non-negative Matrix Factorization (NMF) clustering, Weighted Correlation Network Analysis (WGCNA), and machine learning methods to explore the role of nucleotide metabolism in LUAD. It assesses its association with patient clinical features and the immune microenvironment. Through the analysis of single-cell sequencing data, this study further reveals the distribution of nucleotide metabolism feature genes across different cell subpopulations and their potential connections with immune regulation and the modulation of the tumor microenvironment. By integrating a variety of bioinformatics tools and algorithms, we aim to uncover the potential significance and prognostic value of nucleotide metabolism in the development of LUAD and explore its viability as a potential therapeutic target.

## 2 Materials and methods

### 2.1 Data acquisition

We obtained transcriptomic data in Transcripts Per Kilobase per Million mapped reads (TPM) format and corresponding clinical information for 539 lung adenocarcinoma samples and 59 normal samples from The Cancer Genome Atlas (TCGA) (access link: https://portal.gdc.cancer.gov/). We selected samples with a final diagnosis of lung adenocarcinoma and complete prognostic information (patients with a survival time not equal to 0 and a clearly defined survival status at the end of follow-up). Consequently, a total of 503 tumor samples were included in the subsequent analysis.

Clinical data and somatic mutation data for each patient were also downloaded (access link: https://portal.gdc.cancer.gov/). The Tumor Immune Dysfunction and Exclusion (TIDE) scores for this cohort were obtained from the TIDE website (access link: http://tide.dfci.harvard.edu/), and the Immune Phenotype Scores (IPS) were sourced from The Cancer Immunome Atlas (TCIA) database (access link: https://tcia.at/home) (5–7). We collected 28 gene sets related to nucleotide metabolism from the Molecular Signatures Database (MSigDB) on the GSEA website (access link: https://www.gsea-msigdb.org/gsea/msigdb/index.jsp) (8, 9). The single-cell sequencing dataset GSE207422, based on the GPL24676 platform (Illumina NovaSeq 6000, Homo sapiens), includes 15 non-small cell lung cancer samples pre- and post-immunotherapy combined with chemotherapy, and was sourced from the Gene Expression Omnibus (GEO) database (10).

### 2.2 NMF clustering for nucleotide metabolism subtypes

Expression profiles for 1070 genes associated with nucleotide metabolism were analyzed using non-negative matrix factorization (NMF) clustering. The NMF technique, alongside the ‘brunet’ method, was applied to categorize the samples. The cluster number (K) was varied from 2 to 10 to determine the best fit, ascertained through metrics such as cophenetic correlation, dispersion, and silhouette scores.

### 2.3 Weighted correlation network analysis

The ‘WGCNA’ R package was employed to analyze protein-coding genes in 503 LUAD samples (11). During this process, an appropriate power exponent was selected to convert the adjacency matrix (AM) into a topological overlap matrix. The cut height was set at 100,000, and the R2 at 0.9. A gene consensus module correlation matrix with phenotypes was established, selecting nucleotide metabolism subtypes, survival time, survival status, and tumor stage as associated phenotypes. Subsequently, modules significantly related to the phenotype were identified, and nucleotide metabolism genes were intersected with genes contained in selected modules for further analysis.

### 2.4 Machine learning for selecting nucleotide metabolism feature genes

Four machine learning algorithms (KNN, LogitBoost, RF, SVM), based on the R packages ‘caret’, ‘randomForest’, and ‘xgboost’, were used to further select feature genes predictive of nucleotide metabolism subtypes (12). Importance feature was employed for gene importance ranking.

### 2.5 PCA and PCA composite score

Based on the screened nucleotide feature gene expression, PCA dimension reduction was performed on LUAD samples using the ‘psych’ R package, and a principal component score matrix was calculated. Samples were grouped based on their principal component composite score NMRS, using the median.

### 2.6 Immune cell scoring and somatic mutations

Various immune scoring algorithms, based on the R packages ‘CIBERSORT’ and ‘immunedeconv’, were employed to calculate the relative abundance of various types of immune cells in each sample (13, 14). The ESTIMATE algorithm was used to assess tumor microenvironment scores (15). The TIDE algorithm evaluated the immune escape index of each sample, while the IPS algorithm estimated the IPS score of each sample and the potential immune response to PD-L1 and CTLA-4 immune checkpoint inhibitors.

### 2.7 Single-cell analysis

The 10x single-cell dataset GSE207422 was processed using the R package ‘Seurat’ (16). Cell filtering criteria included: gene count per cell greater than 500, mitochondrial gene percentage less than 20%, ribosomal gene percentage less than 50%, and housekeeping gene UMI value greater than or equal to 1. After data normalization, functions from Canonical Correlation Analyses (CCA) - ‘SelectIntegrationFeatures’, ‘FindIntegrationAnchors’, and ‘IntegrateData’ - were used for data integration, followed by centering. Clustering analysis was conducted using the ‘FindClusters’ function in Seurat, with R package ‘umap’ for dimensionality reduction. Cell types were annotated using a combination of automated annotation and manual labeling, with marker genes identified for each cell cluster as follows: B cell (‘CD79A’, ‘CD19’, ‘MS4A1’, ‘IGHM’), Epithelium (‘EPCAM’, ‘KRT19’, ‘KRT8’, ‘KRT7’), Stromal cell (‘COL1A2’, ‘DCN’, ‘COL6A2’, ‘VWF’), Mast cell (‘CPA3’, ‘MS4A2’, ‘KIT’), Myeloid cell (‘LYZ’, ‘MARCO’, ‘C1QB’), Neutrophil (‘FCGR3B’, ‘CXCR2’, ‘S100A8’, ‘S100A9’), NK cell (‘KLRD1’, ‘KLRF1’, ‘CD8A’), pDC (‘CLEC4C’, ‘LILRA4’, ‘IL3RA’), Plasma cell (‘IGHG1’, ‘JCHAIN’, ‘MZB1’), T cell (‘CD3E’, ‘TRBC2’, ‘TRAC’, ‘CD2’). The enrichment scores for nucleotide metabolism feature genes in different cell subpopulations were calculated using the R package ‘AUCell’ (17).

### 2.8 Gene set enrichment analysis

Gene Set Enrichment Analysis (GSEA) was conducted using the R package ‘clusterProfiler’ (18). The ‘org.Hs.eg.db’ package was used for gene ID conversion. The parameter ‘pvalueCutoff’ was set to 0.1, and ‘pAdjustMethod’ to “BH”. Functions or pathways with an adjusted p-value less than 0.05 were considered to have significant enrichment.

### 2.9 Cell culture

The LUAD cell lines, A549 (Cat No. SNL-257, Sunncell), H1975 (Cat No. SNL-087, Sunncell) and LLC (Cat No. SNL-119, Sunncell), have undergone rigorous authentication procedures, including short tandem repeat (STR) analysis, to ensure their authenticity and reliability. The cell lines were cultured in RPMI-1640 or DMEM medium (Gibco, USA), and enhanced with 10% fetal bovine serum (FBS) (Cat No. AC03L055, Shanghai Lifei Lab Biotech, China). Additionally, the cultures were supplemented with 1% antibiotics. The incubation process occurred at a constant temperature of 37 °C in a controlled environment of 5% CO_2_.

### 2.10 Western blotting

As previously reported, cells were lysed in RIPA on ice for 30 min, and then the lysate supernatant was collected by centrifugation and used to determine protein concentration using the BCA assay. The protein sample obtained shall undergo electrophoresis on a 10% SDS-PAGE gel for analysis. Subsequently, the target protein on the SDS-PAGE gel was transferred onto a 0.45 μm PVDF membrane using a blotting experiment. The overnight incubation procedure entailed the utilization of primary antibodies specific to AUNIP (Cat No. bs-15019R, Bioss, dilution 1: 1000), GAPDH (Cat No. 10494–1-AP, Proteintech, dilution 1: 10000), and β-actin (Cat No. 20536–1-AP, Proteintech, dilution 1: 5000). Subsequently, incubation of secondary antibodies was performed, followed by exposure in a darkroom utilizing ECL. The ensuing images were then analyzed for grayscale intensity via Image J software.

### 2.11 RT-qPCR

The extraction of total RNA from cells was carried out utilizing the TRIzol reagent (Cat No. AG21102, Accurate Biotechnology, Hunan, China). RT-qPCR was conducted through the utilization of a reverse transcription kit (Cat No. AG11728, Accurate Biotechnology, Hunan, China). Subsequently, PCR was executed employing a PCR kit (Cat No. AG11701, Accurate Biotechnology, Hunan, China) for the purpose of amplifying the cDNA generated during the reverse transcription process. All values were normalized relative to their respective β-actin values, and the quantification of fold change was performed using the 2^-ΔΔCt^ method.

The sequence of primers for RT-qPCR as follows:

h-AUNIP forward: 5′-GCGGAAAGTGCAGACACATTT-3′;

h-AUNIP reverse: 5′-TCTCTGGTGAATGCCTGTAGAT-3′.

h-β-actin forward: 5′-AAAGACCTGTACGCCAACAC-3′;

h-β-actin reverse: 5′-GTCATACTCCTGCTTGCTGAT-3′.

### 2.12 Immunohistochemistry

Following the embedding of the LUAD tissue paraffin blocks, each with a thickness approximating 1 mm, the tissue sections were meticulously adhered to the slides. Subsequently, the tissues underwent the processes of deparaffinization and dehydration. After epitope retrieval, H_2_O_2_ treatment, and blocking of non-specific antigens, the LUAD tissues were incubated overnight at a temperature of 4 °C with monoclonal rabbit anti-human AUNIP (Cat No. bs-15019R, Bioss, dilution 1: 200). This was followed by incubation with a secondary antibody, and signal detection was accomplished utilizing a DAB staining kit sourced from Vector Laboratories in the United States. The Histochemistry Score (H-Score = ∑ (PI × I), calculated as the sum of (percentage of cells with weak staining intensity multiplied by 1), (percentage of cells with moderate staining intensity multiplied by 2), and (percentage of cells with strong staining intensity multiplied by 3)) was determined using the Quant Center Analysis tool. The staining intensity was objectively assessed through blind scoring.

### 2.13 Cell counting Kit−8 assay

The LUAD cells inoculated into 96 - well plates with ~1 × 10^4^ cells/well and cultured at 37°C with 5% CO_2_. The experiment will be divided into multiple treatment groups, repeated ≥ 3 times. During culture, 10 μL of CCK-8 reagent (Cat No. C0037, Beyotime, China) will be added to each well and incubated for 1 h. Absorbance values will be measured at 450 nm using a spectrophotometer to calculate proliferation under different interventions. Monitoring will last for 5 days.

### 2.14 Transwell and colony formation assay

After the intervention, cells were divided into treatment groups. Cells diluted with serum-free medium were added to the upper chamber of a 24 - well Transwell plate (2 × 10^4^ cells per well), while the lower chamber received 500 μL of 8% fetal bovine serum medium. The cells were cultured in a 37°C, 5% CO_2_ incubator for 24 h. On the second day, the cells were washed with PBS, fixed with methanol, stained with 0.1% crystal violet, and washed again with PBS. Finally, the microscopy-based cell counting was conducted.

Cells were seeded onto 6-well plates at approximately 1000 cells per well and incubated in a cell culture incubator. The cell culture medium was replaced every 2 days. In 14 days, wash the cells in a 6 - well plate once with PBS. Soak the cells in paraformaldehyde fixative for 30 min. Then stain the cells with crystal violet for 20 min. After washing twice with distilled water, let them dry and take photos.

### 2.15 Subcutaneous tumor formation in mice

Six-week-old male C57BL/6j mice were purchased from Hunan SJA Laboratory Animal Co., Ltd. (Hunan, China). LLC cells (stably transfected with shControl/shAUNIP 1#) in 100 μL PBS were injected subcutaneously into the right back of each mouse (5 × 10^6^ cells per mouse). The length (L) and width (W) of the transplanted tumors were measured every 3 days, and the subcutaneous tumor volumes were determined by the formula of volume = L × W^2^/2. Mice were euthanized after 15 days, and the transplanted tumors were weighed and then subjected to immunofluorescence.

### 2.16 Statistical analysis

All tasks pertaining to data processing, statistical analysis, and visualization were executed utilizing R software, specifically version 4.2.0. The Kaplan-Meier method, alongside the log-rank test, were utilized to estimate and compare subtype-specific overall survival rates. Depending on the distribution of the data, either the analysis of variance (ANOVA) was employed to assess differences in continuous variables across groups. Categorical variables were analyzed using either the chi-square test or Fisher’s exact test. Additionally, Spearman’s correlation analysis was conducted to determine correlations among variables. All p-values were computed using a two-tailed approach, with a statistical significance threshold set at *p* < 0.05.

## 3 Results

### 3.1 Nucleotide metabolism subtypes and prognosis in lung adenocarcinoma

**Figure 1:**
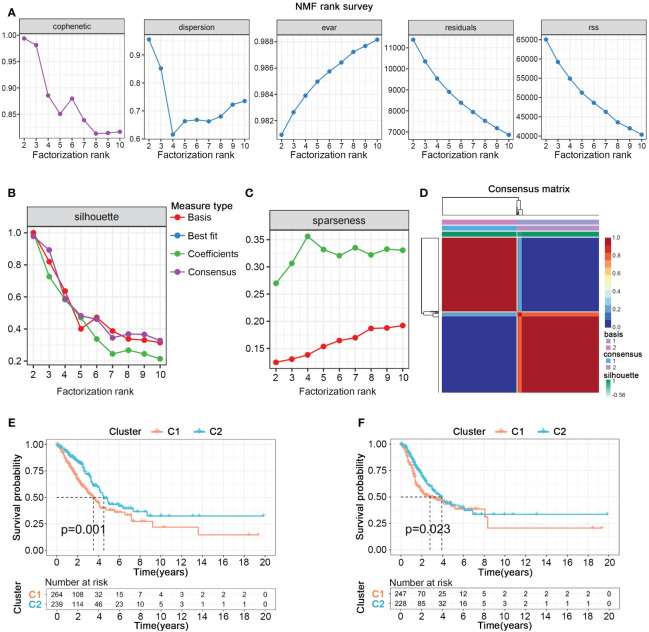
Nucleotide metabolism subtypes and prognosis in LUAD. **(A–C)** Cophenetic distributions, residual sum of squares (RSS), and dispersion indices for ranks 2–10. **(D)** Consensus map from non-negative matrix factorization clustering (K = 2). **(E)** Overall Kaplan-Meier survival curves for both subtypes. **(F)** Progression-free Kaplan-Meier survival curves for both subtypes.

We collected gene sets related to nucleotide metabolism from the GSEA database, comprising 1070 genes closely associated with the synthesis, degradation, and transport processes of nucleotides. To further explore the potential pivotal roles of nucleotide metabolism genes in cancer, we employed univariate Cox analysis to select 297 genes significantly correlated with the overall survival (OS) and survival status of 503 patients with LUAD from TCGA. NMF clustering analysis indicated that these 297 genes could distinctly classify all LUAD patients into two different gene expression patterns (**Figures 1A–D**). Kaplan-Meier survival curves showed significant differences in both overall survival (OS) and progression-free survival (PFS) between the two cluster (**Figures 1E, F**).

### 3.2 Crosstalk between nucleotide metabolism subtypes and key metabolic pathways

**Figure 2:**
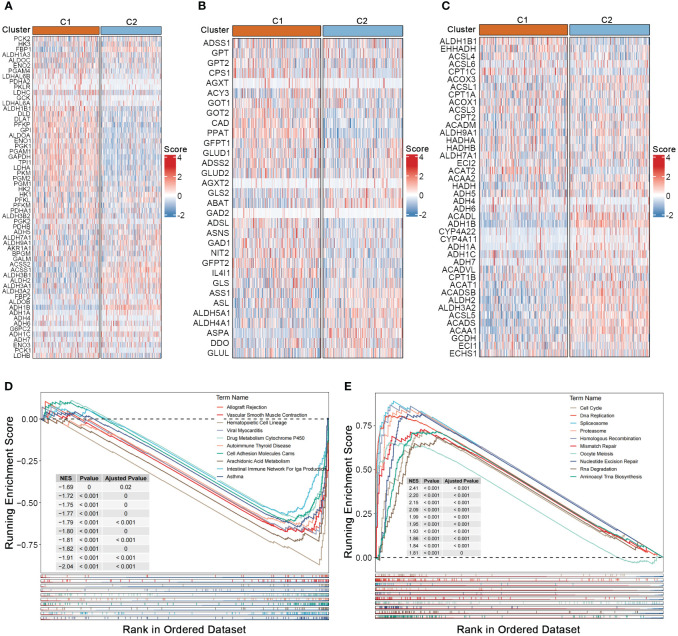
Crosstalk between nucleotide metabolism subtypes and key metabolic pathways. **(A)** Differences in glycolysis-related genes between subtypes. **(B)** Differences in amino acid metabolism-related genes between subtypes. **(C)** Differences in lipid metabolism-related genes between subtypes. **(D)** Gene set enrichment analysis (GSEA) reveals pathways downregulated in subtype C1 relative to C2. **(E)** GSEA reveals pathways upregulated in subtype C1 relative to C2.

To investigate the association between nucleotide gene expression patterns and genes related to carbohydrate, lipid, and amino acid metabolism, we gathered gene sets related to these three major metabolic processes from the GSEA database. Significant metabolic differences were observed between the two nucleotide metabolism subtypes; genes associated with glucose transport and gluconeogenesis, such as LDHA and LDHB, as well as most genes involved in amino acid synthesis metabolism, such as GOT1 and GOT2, were significantly upregulated in subtype C1 (**Figures 2A, B**) (19, 20). Conversely, a considerable proportion of lipid metabolism genes such as ALDH gene family, were upregulated in subtype C2, indicating a strong correlation between the three major metabolic processes and nucleotide gene expression patterns, while also suggesting potential crosstalk within the complex regulatory networks of metabolic-related genes (**Figure 2C**) (21). GSEA enrichment analysis showed that pathways such as immune function, drug metabolism and cell adhesion were generally downregulated in subtype C1 (**Figure 2D**). While numerous nucleotide metabolism pathways including cell cycle, DNA replication, alternative splicing, chromosomal homologous recombination, base mismatch, and nucleotide excision repair were significantly upregulated in C1 (**Figure 2E**). This result, linked with the poorer prognosis of the C1 subtype, further supports the notion that active cell cycle and DNA replication pathways, as well as enhanced glycolysis and amino acid synthesis metabolism, tend to indicate a worse tumor phenotype prognosis.

### 3.3 Identifying characteristic genes of nucleotide metabolism subtypes using WGCNA and machine learning

**Figure 3:**
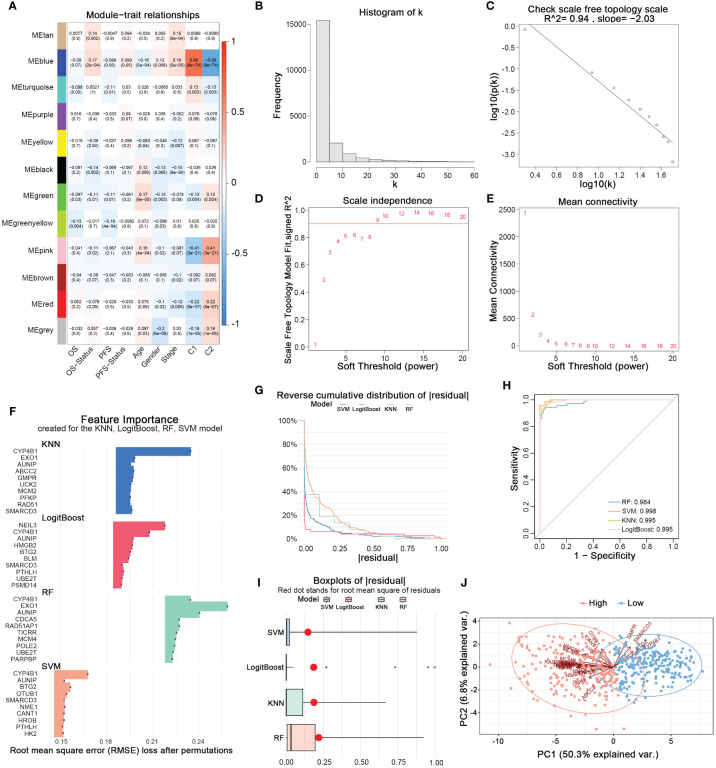
Identifying characteristic genes of nucleotide metabolism subtypes. **(A)** Correlation matrix of WGCNA co-expression modules with various metrics. **(B)** Node frequency in a scale-free network. **(C)** Negative correlation between log(k) and the logarithm of probability [log(p(k))], conforming to the scale-free network topology. **(D)** Correlation between the soft threshold and R^2. **(E)** Mean connectivity under different soft threshold values. **(F)** Top 10 feature genes identified by each machine learning algorithm. **(G)** Reverse cumulative distribution of residuals for four machine learning algorithms. **(H)** The prediction accuracy of nucleotide metabolism subtypes using features identified by different algorithms. **(I)** Root mean square of residuals for different algorithms. **(J)** PCA of lung adenocarcinoma samples based on nucleotide metabolism characteristic genes.

Using the WGCNA method, we established a scale-free topological network matrix of the transcriptomes of 503 LUAD samples, clustering 19,962 mRNAs according to their expression patterns into different gene consensus modules, and finally establishing a correlation matrix between gene consensus modules and clinical information (**Figures 3A–E**). We identified the ‘Blue module’, which was most strongly associated with the clinical staging, survival time, survival status, and nucleotide metabolism subtype of tumor patients, containing 2337 genes. After intersecting with the 297 prognostically relevant nucleotide metabolism genes, we obtained 163 genes. Subsequently, combining KNN, LogitBoost, RF, SVM - four machine learning algorithms, we further selected important feature genes representing the nucleotide metabolism gene expression pattern (**Figures 3F–I**). After merging the top 10 important genes selected by each algorithm and removing duplicates, we obtained 28 feature genes that could predict the metabolic subtype. **Figure 3H** demonstrates that the feature genes selected by each algorithm could distinctly differentiate between the two nucleotide metabolism expression patterns. Following PCA dimension reduction and comprehensive scoring, we calculated the PCA score for each sample based on the expression levels of the 28 genes, termed the nucleotide metabolism-related score (NMRS). The samples were divided into two groups based on the median NMRS, and **Figure 3J** shows that the two groups exhibit strong dissimilarity.

### 3.4 Associations between NMRS scores, clinical features, and oncogenic pathways in lung adenocarcinoma

**Figure 4:**
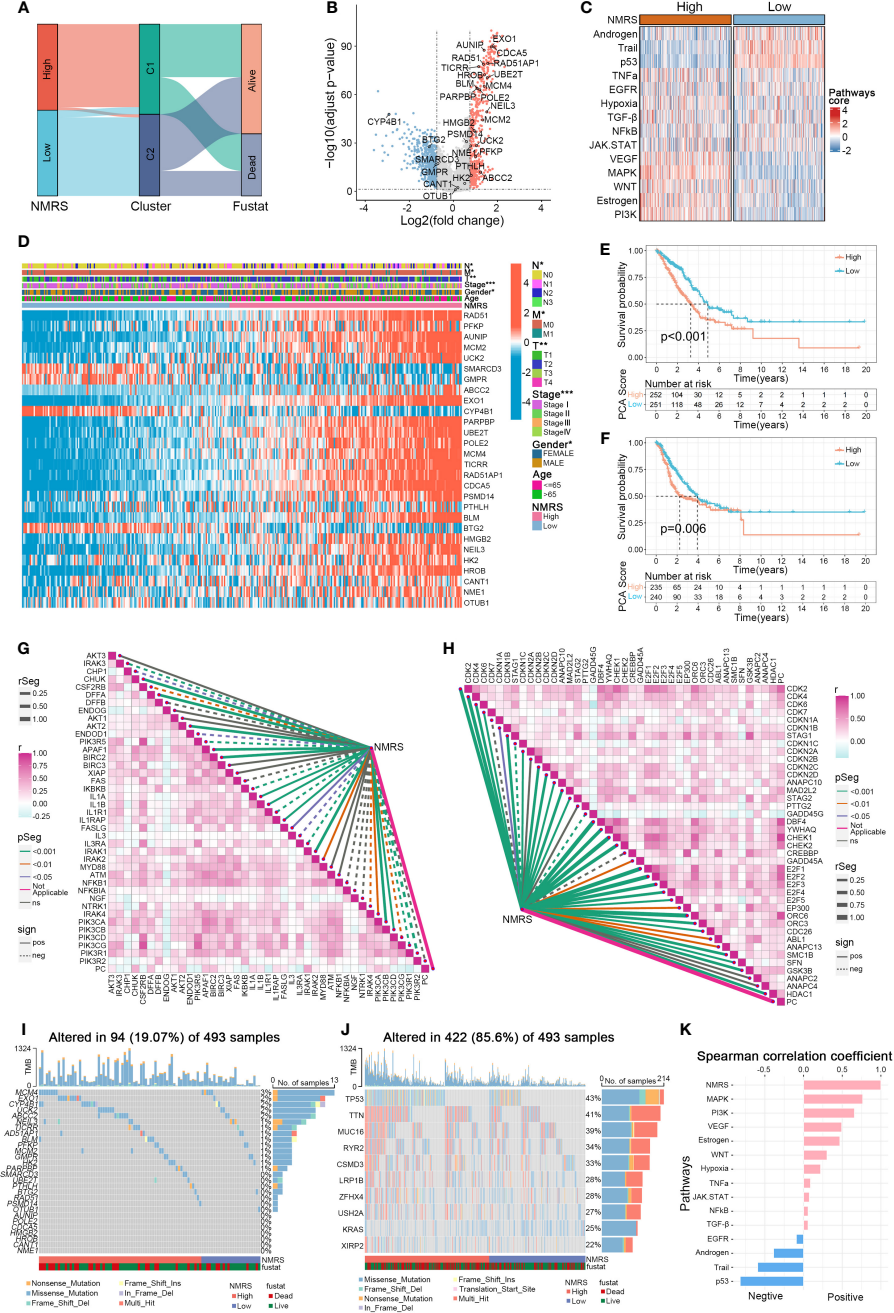
Associations between NMRS scores, clinical features, and oncogenic pathways in LUAD. **(A)** Distribution of NMRS groups among nucleotide metabolism subtypes and survival samples. **(B)** Differential genes between nucleotide metabolism subtypes. **(C)** Activity differences in classic cancer-related pathways between nucleotide metabolism subtypes. **(D)** Relationships between NMRS and various clinical characteristics and gene expression levels. **(E)** Overall Kaplan-Meier survival curves for high and low NMRS groups. **(F)** Progression-free Kaplan-Meier survival curves for high and low NMRS groups. **(G)** Correlation of NMRS with apoptosis-related genes. **(H)** Correlation of NMRS with cell proliferation-related genes. **(I)** Frequency of characteristic gene mutations among different nucleotide metabolism subtypes in lung adenocarcinoma patients. **(J)** Distribution of the top 10 genes with the highest mutation frequencies across different subtypes. **(K)** Correlation of NMRS with enrichment scores of different classic tumor pathways.

Panel A shows that C1 samples are concentrated in the high NMRS group, while C2 samples are predominantly in the low NMRS group (**Figure 4A**). The 28 genes can be broadly categorized into oncogenes and tumor suppressor genes based on previous literature, with oncogenes such as ABCC2 and PFKP upregulated in the C1 subtype, and tumor suppressor genes like BTG2 downregulated in C1 (**Figure 4B**). The function of some genes in lung cancer remains unclear, with AUNIP, notable for its significant p-value, warranting further validation in LUAD cell lines. Subsequently, using the R package ‘decoupleR’, we calculated the scores of classical cancer pathways for each sample (**Figures 4C, K**). The high NMRS group exhibited upregulation of oncogenic pathways such as EGFR, VEGFR, MAPK, PI3K, in contrast to the significant suppression of tumor-suppressor pathways like p53, Androgen, and Trail. This aligns with our earlier results linking nucleotide metabolism subtype C1 to a worse cancer phenotype prognosis. Moreover, we observed that patients with a high NMRS score tend to present with later tumor stages, and the NMRS score is significantly correlated with T, M, N stages, and gender (**Figure 4D**). Significant differences in overall survival (OS) and progression-free survival (PFS) were observed between patients in the high and low NMRS groups (**Figures 4E, F**). We then examined the relationship between the nucleotide metabolism score and genes related to tumor apoptosis and the cell cycle. Interestingly, the NMRS showed a negative correlation with most apoptosis-related genes and a positive correlation with most cell cycle-related genes, indicating that tumors with a higher NMRS score are more inclined to resist apoptosis and engage in more active cell cycle replication processes (**Figures 4G, H**). Compared to LUAD patients with high mutation frequency in TP53 and TTN genes, these nucleotide feature genes generally had a lower overall mutation frequency in the genome and were observed to have a higher mutation frequency in the high NMRS group (**Figures 4I, J**).

### 3.5 NMRS as a potential predictor of microenvironment and immunotherapy response

**Figure 5:**
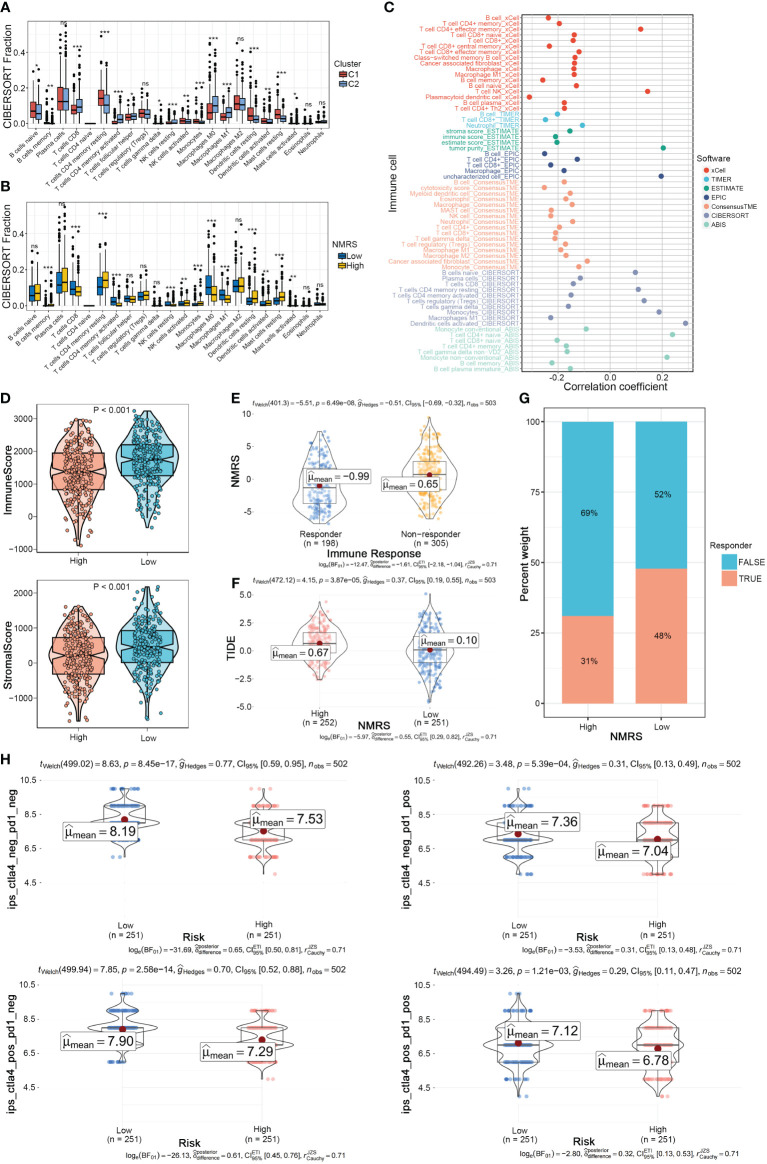
NMRS as a scoring criterion for the immune microenvironment. **(A, B)** Differences in infiltration levels of 22 immune cell types between nucleotide metabolism subtypes and between NMRS groups. **(C)** Correlation of NMRS with various immune cells as revealed by seven different algorithms. **(D)** Differences in tumor microenvironment scores between different NMRS groups as revealed by the ESTIMATE algorithm. **(E)** Differences in NMRS scores between populations responding and not responding to immunotherapy as predicted by the TIDE algorithm. **(F)** Differences in TIDE scores between different NMRS groups. **(G)** Distribution of immunotherapy beneficiaries among different NMRS groups. **(H)** Differences in IPS scores predicting effectiveness of PD-L1 or CTLA-4 inhibitor treatments between different NMRS groups.

We further explored the differences in the tumor microenvironment between NMRS groups. First, using the CIBERSORT algorithm for deconvolution, we obtained the relative abundance of 22 types of immune cells. We observed a high degree of consistency between nucleotide metabolism subtypes and NMRS grouping in the tumor microenvironment. Immune cells such as CD8+ T cells, activated memory CD4+ T cells, and Macrophages M0/M1 were significantly upregulated in the C2 type and low NMRS group, while Macrophages M2, Dendritic cells, etc., were significantly upregulated in the C1 type and high NMRS group, indicating that patients with C2 type and low NMRS group tend to have a more active tumor microenvironment (**Figures 5A, B**). Seven tumor microenvironment and immune cell calculation methods further supported this conclusion, with NMRS scores showing a significant negative correlation with the majority of immune cell contents calculated by various software systems, while showing a high positive correlation with suppressive immune cells, epithelial cells, and tumor purity (**Figure 5C**). The ESTIMATE algorithm indicated that patients in the low NMRS group have higher overall levels of immune cells, suggesting that LUAD patients with a low NMRS score may have a better immune therapy response (**Figure 5D**). The TIDE algorithm showed that patients in the high NMRS group have higher TIDE scores, indicating a higher possibility of immune escape and poorer immune therapy response in this group (**Figure 5E**). Correspondingly, TIDE predictions indicate a lower NMRS score in populations with an immune response, with a higher proportion of patients in the low NMRS group responding to the immune response (**Figures 5F, G**). The IPS score supports this result, with the IPS scoring system predicting that patients with a low NMRS score have higher IPS scores when using PD-L1 and CTLA-4 immune checkpoint inhibitors, implying a stronger potential therapeutic effect (**Figure 5H**).

### 3.6 Single-cell analysis of NMRS in LUAD pre- and post-immunochemotherapy

**Figure 6:**
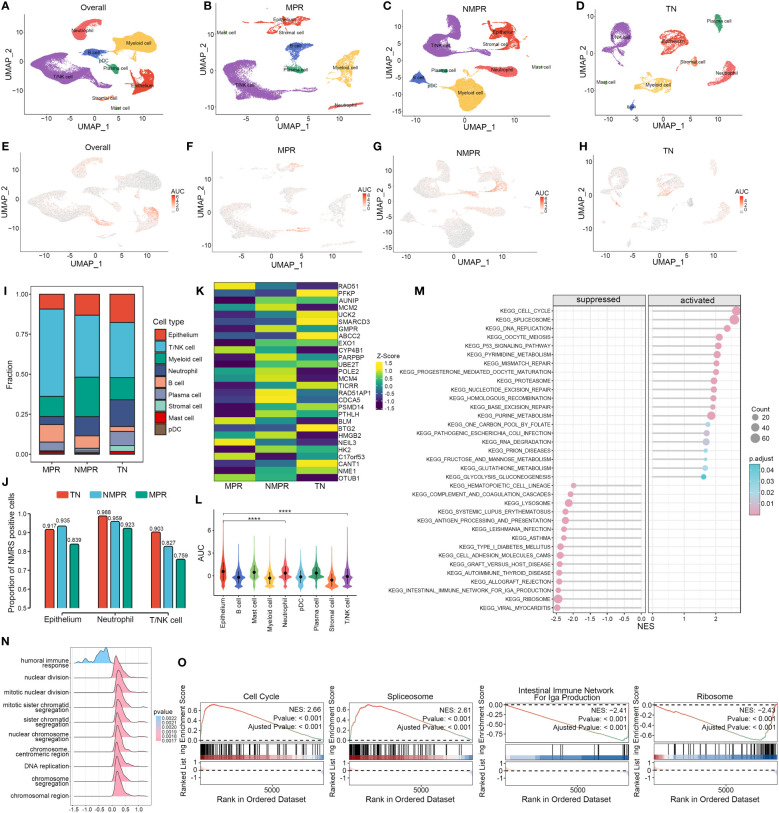
Single-cell analysis of NMRS in LUAD pre- and post-immunochemotherapy. **(A–D)** Single-cell analysis revealed the differences in cell subpopulations across all patients **(A)**, the MPR group **(B)**, the NMPR group **(C)**, and the TN group **(D)**. **(E–H)** The ‘AUCell’ algorithm revealed the differences in cell distribution of NMRS signature genes across different groups. **(I)** Differences in the abundance of cell types across different groups. **(J)** The differences in the abundance of NMRS signature genes among epithelial cells, T/NK cells, and neutrophils across different groups. **(K)** The transcriptional level differences of 28 NMRS signature genes. **(L)** The differences in the abundance of NMRS signature genes across different cell subpopulations in all patients. **(M)** Enriched pathways in cells with high versus low AUC scores. **(N)** Functional differences between cells with high and low AUC scores. **(O)** GSEA reveals significantly altered pathways in cells with high AUC scores compared to those with low scores.

We explored the distribution of NMRS marker genes in GSE207422. This dataset aims to explore changes in the tumor microenvironment (TME) of non-small cell lung cancer (NSCLC) following targeted PD-1 immunotherapy combined with chemotherapy. Single-cell sequencing was performed on samples from 3 patients before treatment and 12 patients after receiving the combined therapy. Based on pathological response, the 12 post-treatment samples were divided into two groups: the major pathologic response (MPR) group (n = 4) and the non-major pathologic response (NMPR) group (n = 8). After excluding non-qualifying cells, a total of 92,003 cells were included in the subsequent analysis. Based on the marker genes of various cell types, we annotated the cells into nine distinct categories (**Figures 6A–D**). Compared to the treatment-naive (TN) group, the MPR and NMPR groups exhibited an increase in T/NK cells and B cells, while tumor epithelial cells decreased (**Figure 6I**). Using the ‘AUCell’ algorithm, we observed that there was no significant change in the distribution trend of NMRS characteristic genes across the TN, NMPR, and MPR groups. These genes predominantly localized within the epithelium, T/NK cells, and neutrophils both before and after treatment (**Figures 6E–H, L**). Interestingly, the relative abundance of NMRS in these three cell types significantly decreased in the MPR group (**Figure 6J**). Additionally, we found that the expression levels of NMRS characteristic genes were generally lower in the MPR group compared to the TN group, whereas the NMPR group showed a relatively higher abundance of these genes (**Figure 6K**).

Based on the GSEA algorithm, cells with high AUC scores in the total malignant epithelial cell subgroup significantly upregulated functions such as DNA replication, chromosomal reconstruction, and cell nuclear division, while immune-related functions were significantly downregulated (**Figure 6N**). Pathway analysis indicated that various key nucleotide metabolism pathways, such as cell cycle, alternative splicing, DNA replication, were significantly upregulated in cells with high AUC scores, while pathways like ‘Intestinal immune network for IgA production’ and ‘RIBOSOME’ were significantly downregulated (**Figures 6M, O**).

### 3.7 AUNIP promotes LUAD cell proliferation and modulates immune infiltration

**Figure 7:**
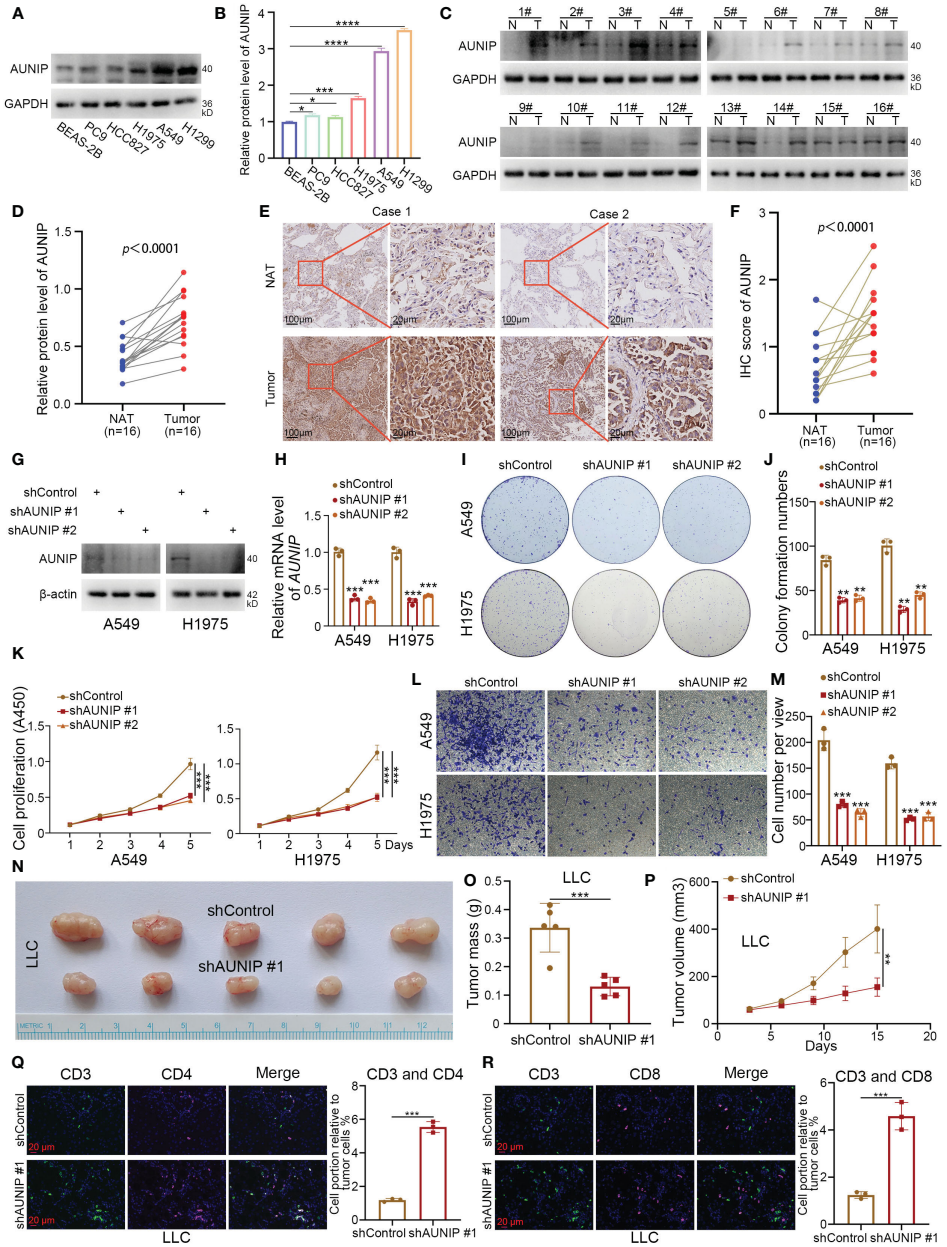
AUNIP As an oncogene promoting the progression of LUAD. **(A, B)** The lung normal or LUAD cell lines were harvested for Western blot analysis. Data presents as mean ± SD with three replicates. *p < 0.05; ***p < 0.001; ****p < 0.0001. **(C, D)** The protein expression levels of AUNIP in the adjacent non-tumor lung tissues and LUAD tissues were analyzed by the western blot. The protein levels of AUNIP were quantified by the image J software. *p* value as indicated. **(E, F)** The IHC staining was performed in the LUAD and normal lung tissues by using the AUNIP antibody. *p* value as indicated. **(G–M)** A549 and H1975 cells were infected with shControl, shAUNIP #1, or shAUNIP #2 for 72 h. Cells were collected for Western blot analysis **(G)**, RT-qPCR analysis **(H)**, colony formation assay **(I, J)**, CCK-8 assay **(K)**, Transwell assay **(L, M)**. Data presents as mean ± SD with three replicates. **p < 0.01; ***p < 0.001. **(N-R)** LLC cells were transfected with the described shRNAs for 72 h. Cells were collected and injected subcutaneously into the C57 mice. The tumors were removed **(N)** and measured for mass **(O)**, growth curve **(P)**, and immunofluorescence **(Q, R)**. Data presents as mean ± SD with five or three replicates. **p < 0.01; ***p < 0.001.

After intersecting the top 10 genes predicted by four machine learning methods for subtype identification, only AUNIP and CYP4B1 remained. This indicates that AUNIP may be one of the most universally predictive genes. Additionally, the strong positive correlation between AUNIP expression levels and NMRS, along with its positive correlation with most NMRS characteristic genes, suggests AUNIP may not only serve as a classification marker for NMRS subtypes but also reveal its potential pro-oncogenic role in lung adenocarcinoma (**Supplementary Figures 2A, I**). Therefore, we have chosen to validate the biological function of AUNIP in lung adenocarcinoma. To investigate the expression level of AUNIP in LUAD tumors. The results of the Western blotting experiment demonstrated that the expression level of AUNIP is higher in LUAD cell lines compared to the normal alveolar epithelial cell line BEAS-2B (**Figures 7A, B**). Later, we discovered that the expression of AUNIP in LUAD tumor tissue was higher than in the adjacent non-cancerous tissue (**Figures 7C, D**). This finding was further validated through immunohistochemical staining (**Figures 7E, F**). Additionally, we knocked down the level of AUNIP in LUAD cells by transfecting shAUNIP (**Figures 7G, H**). Interestingly, we observed that knocking down AUNIP in LUAD cells significantly reduced their clonogenic and proliferative capabilities (**Figures 7I–K**). Furthermore, the TRANSWELL results demonstrate that silencing AUNIP can reduce the migratory capacity of LUAD cells (**Figures 7L, M**).

In our research, based on the ‘ESTIMATE’ and deconvolution algorithms, AUNIP shows a significant positive correlation with tumor purity scores, and a negative correlation with immune scores, B cells, CD8+ T cells, and CD4+ T cells in the lung adenocarcinoma cohort from the TCGA database (**Supplementary Figures 2C–H**). Ma et al. also demonstrated that the expression of AUNIP is associated with immune infiltration in hepatocellular carcinoma (HCC) and LUAD (22). Next, we performed subcutaneous tumorigenicity experiments by LLC cells in C57 mice, and the results showed that knocking down AUNIP inhibited cell proliferation *in vivo* (**Figures 7N–P**). In addition, we found that knockdown of AUNIP increased the degree of infiltration of CD3+ CD4+ T cells and CD3+ CD8+ T cells in tumors (**Figures 7Q, R**). Thus, our experimental results suggest that AUNIP acts as an oncogene to promote the proliferation and migration of LUAD cells and correlates with the immune infiltration of T cells in LUAD.

## 4 Discussion

Lung cancer has become a major challenge in the field of global public health, causing a heavy burden on the global economy every year (23). Although there has been some progress in the treatment of lung cancer, the therapeutic effect still fails to meet expectations, seriously violating the rights and interests of patients’ lives and health (24). In order to more effectively evaluate the prognosis of lung cancer patients, it is particularly important to develop reliable predictive tools. Nucleotide metabolism, as a basic metabolic process closely related to tumor growth, plays a key role in cancer progression (25). Studies have shown that nucleotide metabolism has the potential to become a new indicator for evaluating the prognosis of lung cancer patients, and is expected to provide more scientific and accurate basis for clinical decision-making (26).

In this study, we conducted a Cox analysis by employing the coxph function in R, along with NMF algorithm based on nucleotide metabolism-related genes. Using this approach, we successfully identified two distinct clusters of LUAD. Furthermore, we characterized the gene expression profiles within each cluster.

The poor prognosis associated with tumor phenotypes is often closely related to the abnormally active cell cycle, overactive DNA replication pathways, as well as enhanced glycolysis and amino acid synthesis metabolism (27–29). These biological processes play crucial roles in the development and progression of tumors, which not only promote rapid proliferation and metastasis of tumor cells but also increase the challenges of treatment and the risk of poor prognosis (30). Interestingly, we discovered that the upregulated genes in subtype C1 are primarily associated with glucose transport, gluconeogenesis, and amino acid anabolic metabolism, key metabolic processes. On the other hand, subtype C2 has a focus on lipid metabolism. These findings unveil a strong correlation between nucleotide gene expression patterns and the three metabolic processes mentioned. Furthermore, upon performing GSEA enrichment analysis, we observed a significant upregulation of various nucleotide metabolism pathways in C1 compared to C2. These pathways encompass cell cycle, DNA replication, alternative splicing, homologous recombination, base mismatch repair, and nucleotide excision repair, among others. Additionally, we identified a general downregulation trend in genes associated with drug metabolism and cell adhesion pathways in the C1 subtype.

Next, we employed WGCNA to establish co-expression classification and identified genes significantly associated with clustering. Ultimately, we identified the “blue module”, a gene consensus module that is most correlated with clinical staging, survival time, survival status, and nucleotide metabolism subtypes in tumor patients. Moreover, by integrating four machine learning algorithms: KNN, LogitBoost, RF, and SVM, we identified 28 characteristic genes capable of predicting metabolic subtypes. Upon further analysis using PCA dimensionality reduction and a comprehensive scoring system, our findings revealed that C1 samples were primarily concentrated in the high NMRS group, whereas C2 samples were predominantly grouped in the low NMRS group.

Multiple studies have demonstrated that ABCC2 and PFKP play a carcinogenic role in the development of lung cancer. Additionally, patients with LUAD who have lower expression of the BTG2 gene in their tumor tissue tend to have a poorer prognosis (31–33). Consistent with these findings, our analysis revealed that in C1 samples, the expression levels of ABCC2 and PFKP were upregulated, while tumor suppressor genes such as BTG2 were downregulated. Subsequently, the R package ‘decoupleR’ was utilized to calculate the canonical cancer pathways for each sample. The results demonstrated that oncogenic pathways such as EGFR, VEGFR, MAPK, and PI3K were upregulated in the high NMRS group, whereas tumor suppressor pathways including p53, Androgen, and Trail exhibited significant suppression (34). Moving forward, we conducted a deeper analysis to explore the correlation between NMRS scores and T, M, N staging, as well as gender. Interestingly, we also observed that patients with higher NMRS scores tended to exhibit later tumor staging. Additionally, patients in the high NMRS group exhibited shorter overall survival (OS) and progression-free survival (PFS). Furthermore, our analysis indicates that tumors in the high NMRS group exhibit a more active cell cycle and possess stronger anti-apoptotic capabilities. These analytical results further corroborate the association between C1 nucleotide metabolism subtypes and the poor prognosis of LUAD cancer phenotypes.

The tumor microenvironment serves as one of the key driving factors for various malignant tumors (35). Previous studies have established a correlation between CD8A and the existence of CD8+ T cells, which exhibit anti-tumor activities, thereby suggesting more favorable immunotherapy outcomes (36). Conversely, PD-L1, an immune checkpoint protein, is associated with tumor cells evading immune responses, potentially resulting in poorer prognoses for patients (37). Therefore, we analyzed the relative abundance of 22 immune cell types using the CIBERSORT algorithm. The analysis reveals a strong correlation between nucleotide metabolism subtypes and NMRS groupings with the tumor microenvironment, where patients with subtype C2 and low NMRS scores have a more active tumor microenvironment. Further analysis using seven tumor microenvironment and immune cell calculation methods showed a significant negative correlation between NMRS scores and the majority of immune cell populations, but a strong positive correlation with suppressive immune cells, epithelial cells, and tumor purity. Furthermore, the results of ESTIMATE, TIDE, and IPS scoring suggest that LUAD patients in the high NMRS group have a poorer response to immunotherapy and a higher likelihood of immune evasion. Conversely, LUAD patients in the low NMRS group may be more sensitive to immunotherapy.

The single-cell RNA sequencing (scRNA-seq) is a powerful technique for characterizing the heterogeneity of immune and tumor cells in LUAD (38). The technique enables detailed gene expression analysis at the single-cell level, revealing rare cell populations and intercellular communication within the tumor immune microenvironment (TIME), which may lead to the development of novel therapeutic strategies against LUAD (39). Therefore, a comprehensive analysis was conducted on the distribution patterns of NMRS marker genes within a comprehensive dataset of resectable non-small cell lung cancer patients who underwent a combined immunotherapy and chemotherapy treatment protocol. It is well known that targeted immunotherapy of PD-L1 influence on immune cells within the tumor microenvironment is extremely important (40, 41). Of interest, studies have shown that targeting nucleotide metabolism enhances anti-tumor immune responses (26). Therefore, we selected the single-cell sequencing dataset GSE207422 for analysis to explore the changes in the distribution of NMRS marker genes in NSCLC patients before and after targeted PD-1 immunotherapy combined with chemotherapy. Before and after treatment, there was a significant decrease in malignant epithelial cells and an increase in the proportion of immune cells such as T/NK cells, which is consistent with clinical practice. The distribution characteristics of the major cell types of the nucleotide metabolism signature genes did not show significant changes, mainly malignant epithelial cells, followed by neutrophils and T/NK cells, and this relatively fixed localization implies that the NMRS signature genes may be characterized by both malignant tumor biology and immune-related functions. Interestingly, the transcript levels of most NMRS-characterized genes showed a general down-regulation in the MPR group, while the NMPR group showed insignificant down-regulation or even up-regulation, such as AUNIP and CDCA5. Comparing the TN and NMPR groups, the overall NMRS abundance in the MPR group was significantly decreased in malignant epithelial cells, neutrophils and T/NK cells. Notably, there is a significant upregulation of various nucleotide metabolism pathways, including cell cycle, alternative splicing, and DNA replication, specifically in the high-NMRS group of malignant epithelial cells. Conversely, a downregulation is observed in the IgA production of the intestinal immune network and the ribosome pathway. Thus, these results suggest that patients in the low NMRS group have a higher sensitivity to targeted PD-1 immunotherapy combined with chemotherapy, possibly through direct or indirect targeting of relevant genes characterized predominantly by nucleotide metabolism.

AUNIP (Aurora kinase A and ninein-interacting protein) plays a crucial role in preserving the integrity of the centrosomal structure and promoting spindle assembly (42). The study found a correlation between AUNIP and immune and stromal scores in oral squamous cell carcinoma (OSCC), indicating a potential role in recruiting infiltrating immune and stromal cells in the tumor microenvironment of OSCC (43). Interestingly, Ma et al. found that AUNIP is a potential diagnostic and prognostic biomarker for liver cancer and lung cancer (22). While the precise biological function of AUNIP in LUAD remains enigmatic, our study has identified it as an oncogene that demonstrates elevated expression in high NMRS. Based on the “ESTIMATE” and deconvolution algorithms, our results similarly showed that AUNIP was significantly positively correlated with tumor purity scores, and negatively correlated with immune scores, B-cells, CD8+ T-cells and CD4+ T-cells. In addition, we further showed by *in vivo* and *in vitro* experiments that AUNIP acts as an oncogene to promote the proliferation and migration of LUAD cells and is associated with immune infiltration of T cells.

Our research, despite its significance, is not without limitations. Presently, our model still has numerous risk factors yet to incorporate, thereby limiting its widespread practical application. To address this, we are committed to monitoring advancements in prognostic models and actively incorporating additional risk factors to further enhance and refine our model. Furthermore, recognizing that this study primarily relies on retrospective data, we intend to strengthen our focus on prospective research in the future to derive more precise and comprehensive conclusions.

## 5 Conclusion

Utilizing various databases and analytical techniques, we conducted a thorough investigation to assess the potential significance and prognostic implications of nucleotide metabolism in the progression of LUAD. Furthermore, we explored the feasibility of targeting nucleotide metabolism as a potential therapeutic approach.

## Data availability statement

The datasets presented in this study can be found in online repositories. The names of the repository/repositories and accession number(s) can be found in the article/**Supplementary Material**.

## Ethics statement

The studies involving humans were approved by The Medical Ethics Committee of the Second Xiangya Hospital of Central South University. The studies were conducted in accordance with the local legislation and institutional requirements. The participants provided their written informed consent to participate in this study.

## Author contributions

DL: Data curation, Formal analysis, Methodology, Software, Writing – original draft. HHW: Formal analysis, Methodology, Writing – original draft, Investigation, Validation. ZZ: Investigation, Methodology, Writing – original draft. JC: Methodology, Data curation, Writing – original draft. HQW: Conceptualization, Project administration, Supervision, Writing – review & editing.

## Supplementary material

Supplementary Figure 1
**(A–D)** Thresholds for NMRS signature gene abundance across different groups set by the ‘AUCell’ algorithm.

Supplementary Figure 2
**(A)** Correlation between AUNIP transcription levels and NMRS. **(B–E)**: The ‘ESTIMATE’ algorithm revealed the correlation between AUNIP transcription levels and tumor purity **(B)**, immune score **(C)**, stromal score **(D)**, and the overall ESTIMATE score **(E)**. **(F–H)** Correlation between AUNIP and the relative abundance of three different immune cell types revealed by deconvolution algorithm. **(I)** Correlation of AUNIP transcription levels with the other 27 NMRS signature genes.

## References

[1] Bray F Laversanne M Sung H Ferlay J Siegel RL Soerjomataram I. Global cancer statistics 2022: GLOBOCAN estimates of incidence and mortality worldwide for 36 cancers in 185 countries. CA Cancer J Clin. (2024) 74:229–63. doi: 10.3322/caac.21834 38572751

[2] Horvath L Thienpont B Zhao L Wolf D Pircher A. Overcoming immunotherapy resistance in non-small cell lung cancer (NSCLC) - novel approaches and future outlook. Mol Cancer. (2020) 19:141. doi: 10.1186/s12943-020-01260-z 32917214 PMC7488475

[3] Faubert B Solmonson A Deberardinis RJ. Metabolic reprogramming and cancer progression. Science. (2020) 368(6487). doi: 10.1126/science.aaw5473 PMC7227780 32273439

[4] Rigden DJ Fernández XM. The 2024 Nucleic Acids Research database issue and the online molecular biology database collection. Nucleic Acids Res. (2024) 52:D1–d9. doi: 10.1093/nar/gkad1173 38035367 PMC10767945

[5] Fu J Li K Zhang W Wan C Zhang J Jiang P. Large-scale public data reuse to model immunotherapy response and resistance. Genome Med. (2020) 12:21. doi: 10.1186/s13073-020-0721-z 32102694 PMC7045518

[6] Jiang P Gu S Pan D Fu J Sahu A Hu X. Signatures of T cell dysfunction and exclusion predict cancer immunotherapy response. Nat Med. (2018) 24:1550–8. doi: 10.1038/s41591-018-0136-1 PMC6487502 30127393

[7] Charoentong P Finotello F Angelova M Mayer C Efremova M Rieder D. Pan-cancer immunogenomic analyses reveal genotype-immunophenotype relationships and predictors of response to checkpoint blockade. Cell Rep. (2017) 18:248–62. doi: 10.1016/j.celrep.2016.12.019 28052254

[8] Subramanian A Tamayo P Mootha VK Mukherjee S Ebert BL Gillette MA. Gene set enrichment analysis: a knowledge-based approach for interpreting genome-wide expression profiles. Proc Natl Acad Sci USA. (2005) 102(43):15545–50. doi: 10.1073/pnas.0506580102 PMC1239896 16199517

[9] Liberzon A Birger C Thorvaldsdóttir H Ghandi M Mesirov JP Tamayo P. The Molecular Signatures Database (MSigDB) hallmark gene set collection. Cell Syst. (2015) 1:417–25. doi: 10.1016/j.cels.2015.12.004 PMC4707969 26771021

[10] Hu J Zhang L Xia H Yan Y Zhu X Sun F. Tumor microenvironment remodeling after neoadjuvant immunotherapy in non-small cell lung cancer revealed by single-cell RNA sequencing. Genome Med. (2023) 15:14. doi: 10.1186/s13073-023-01164-9 36869384 PMC9985263

[11] Langfelder P Horvath S. WGCNA: an R package for weighted correlation network analysis. BMC Bioinf. (2008) 9:559. doi: 10.1186/1471-2105-9-559 PMC2631488 19114008

[12] Hengl T Mendes De Jesus J Heuvelink GB Ruiperez Gonzalez M Kilibarda M Blagotić A. SoilGrids250m: Global gridded soil information based on machine learning. PloS One. (2017) 12:e0169748. doi: 10.1371/journal.pone.0169748 28207752 PMC5313206

[13] Newman AM Liu CL Green MR Gentles AJ Feng W Xu Y. Robust enumeration of cell subsets from tissue expression profiles. Nat Methods. (2015) 12:453–7. doi: 10.1038/nmeth.3337 PMC4739640 25822800

[14] Sturm G Finotello F List M. Immunedeconv: an R package for unified access to computational methods for estimating immune cell fractions from bulk RNA-sequencing data. Methods Mol Biol. (2020) 2120:223–32. doi: 10.1007/978-1-0716-0327-7_16 32124323

[15] Yoshihara K Shahmoradgoli M Martínez E Vegesna R Kim H Torres-Garcia W. Inferring tumour purity and stromal and immune cell admixture from expression data. Nat Commun. (2013) 4:2612. doi: 10.1038/ncomms3612 24113773 PMC3826632

[16] Hao Y Hao S Andersen-Nissen E Mauck WM 3rd Zheng S Butler A. Integrated analysis of multimodal single-cell data. Cell. (2021) 184:3573–3587.e29. doi: 10.1016/j.cell.2021.04.048 34062119 PMC8238499

[17] Aibar S González-Blas CB Moerman T Huynh-Thu VA Imrichova H Hulselmans G. SCENIC: single-cell regulatory network inference and clustering. Nat Methods. (2017) 14:1083–6. doi: 10.1038/nmeth.4463 PMC5937676 28991892

[18] Yu G Wang LG Han Y He QY. clusterProfiler: an R package for comparing biological themes among gene clusters. Omics. (2012) 16:284–7. doi: 10.1089/omi.2011.0118 PMC3339379 22455463

[19] Claps G Faouzi S Quidville V Chehade F Shen S Vagner S. The multiple roles of LDH in cancer. Nat Rev Clin Oncol. (2022) 19:749–62. doi: 10.1038/s41571-022-00686-2 36207413

[20] Birsoy K Wang T Chen WW Freinkman E Abu-Remaileh M Sabatini DM. An essential role of the mitochondrial electron transport chain in cell proliferation is to enable aspartate synthesis. Cell. (2015) 162:540–51. doi: 10.1016/j.cell.2015.07.016 PMC4522279 26232224

[21] Liu L Li T Liao Y Wang Y Gao Y Hu H. Triose kinase controls the lipogenic potential of fructose and dietary tolerance. Cell Metab. (2020) 32:605–618.e7. doi: 10.1016/j.cmet.2020.07.018 32818435

[22] Ma C Kang W Yu L Yang Z Ding T. AUNIP expression is correlated with immune infiltration and is a candidate diagnostic and prognostic biomarker for hepatocellular carcinoma and lung adenocarcinoma. Front Oncol. (2020) 10:590006. doi: 10.3389/fonc.2020.590006 33363020 PMC7756081

[23] Leiter A Veluswamy RR Wisnivesky JP. The global burden of lung cancer: current status and future trends. Nat Rev Clin Oncol. (2023) 20:624–39. doi: 10.1038/s41571-023-00798-3 37479810

[24] Chaft JE Rimner A Weder W Azzoli CG Kris MG Cascone T. Evolution of systemic therapy for stages I-III non-metastatic non-small-cell lung cancer. Nat Rev Clin Oncol. (2021) 18:547–57. doi: 10.1038/s41571-021-00501-4 PMC9447511 33911215

[25] Shi DD Savani MR Abdullah KG McBrayer SK. Emerging roles of nucleotide metabolism in cancer. Trends Cancer. (2023) 9:624–35. doi: 10.1016/j.trecan.2023.04.008 PMC10967252 37173188

[26] Wu HL Gong Y Ji P Xie YF Jiang YZ Liu GY. Targeting nucleotide metabolism: a promising approach to enhance cancer immunotherapy. J Hematol Oncol. (2022) 15:45. doi: 10.1186/s13045-022-01263-x 35477416 PMC9044757

[27] Gaillard H García-Muse T Aguilera A. Replication stress and cancer. Nat Rev Cancer. (2015) 15:276–89. doi: 10.1038/nrc3916 25907220

[28] Ganapathy-Kanniappan S Geschwind JF. Tumor glycolysis as a target for cancer therapy: progress and prospects. Mol Cancer. (2013) 12:152. doi: 10.1186/1476-4598-12-152 24298908 PMC4223729

[29] Sivanand S Vander Heiden MG. Emerging roles for branched-chain amino acid metabolism in cancer. Cancer Cell. (2020) 37:147–56. doi: 10.1016/j.ccell.2019.12.011 PMC7082774 32049045

[30] Stine ZE Schug ZT Salvino JM Dang CV. Targeting cancer metabolism in the era of precision oncology. Nat Rev Drug Discov. (2022) 21:141–62. doi: 10.1038/s41573-021-00339-6 PMC8641543 34862480

[31] Wang Z Wu S Chen X Liang J Xue F Cho WC. PFKP confers chemoresistance by upregulating ABCC2 transporter in non-small cell lung cancer. Transl Lung Cancer Res. (2023) 12:2294–309. doi: 10.21037/tlcr PMC10713276 38090515

[32] Chen J Zou L Lu G Grinchuk O Fang L Ong DST. PFKP alleviates glucose starvation-induced metabolic stress in lung cancer cells via AMPK-ACC2 dependent fatty acid oxidation. Cell Discov. (2022) 8:52. doi: 10.1038/s41421-022-00406-1 35641476 PMC9156709

[33] Chen Z Chen X Lu B Gu Y Chen Q Lei T. Up-regulated LINC01234 promotes non-small-cell lung cancer cell metastasis by activating VAV3 and repressing BTG2 expression. J Hematol Oncol. (2020) 13:7. doi: 10.1186/s13045-019-0842-2 31959200 PMC6972004

[34] Yuan M Zhao Y Arkenau HT Lao T Chu L Xu Q. Signal pathways and precision therapy of small-cell lung cancer. Signal Transduct Target Ther. (2022) 7:187. doi: 10.1038/s41392-022-01013-y 35705538 PMC9200817

[35] De Visser KE Joyce JA. The evolving tumor microenvironment: From cancer initiation to metastatic outgrowth. Cancer Cell. (2023) 41:374–403. doi: 10.1016/j.ccell.2023.02.016 36917948

[36] Reina-Campos M Scharping NE Goldrath AW. CD8(+) T cell metabolism in infection and cancer. Nat Rev Immunol. (2021) 21:718–38. doi: 10.1038/s41577-021-00537-8 PMC8806153 33981085

[37] Tumeh PC Harview CL Yearley JH Shintaku IP Taylor EJ Robert L. PD-1 blockade induces responses by inhibiting adaptive immune resistance. Nature. (2014) 515:568–71. doi: 10.1038/nature13954 PMC4246418 25428505

[38] Jovic D Liang X Zeng H Lin L Xu F Luo Y. Single-cell RNA sequencing technologies and applications: A brief overview. Clin Transl Med. (2022) 12:e694. doi: 10.1002/ctm2.694 35352511 PMC8964935

[39] Ding S Chen X Shen K. Single-cell RNA sequencing in breast cancer: Understanding tumor heterogeneity and paving roads to individualized therapy. Cancer Commun (Lond). (2020) 40:329–44. doi: 10.1002/cac2.12078 PMC7427308 32654419

[40] Zhao L Chen X Wu H He Q Ding L Yang B. Strategies to synergize PD-1/PD-L1 targeted cancer immunotherapies to enhance antitumor responses in ovarian cancer. Biochem Pharmacol. (2023) 215:115724. doi: 10.1016/j.bcp.2023.115724 37524205

[41] Liu J Peng X Yang S Li X Huang M Wei S. Extracellular vesicle PD-L1 in reshaping tumor immune microenvironment: biological function and potential therapy strategies. Cell Commun Signal. (2022) 20:14. doi: 10.1186/s12964-021-00816-w 35090497 PMC8796536

[42] Lieu AS Cheng TS Chou CH Wu CH Hsu CY Huang CY. Functional characterization of AIBp, a novel Aurora-A binding protein in centrosome structure and spindle formation. Int J Oncol. (2010) 37:429–36. doi: 10.3892/ijo 20596670

[43] Yang Z Liang X Fu Y Liu Y Zheng L Liu F. Identification of AUNIP as a candidate diagnostic and prognostic biomarker for oral squamous cell carcinoma. EBioMedicine. (2019) 47:44–57. doi: 10.1016/j.ebiom.2019.08.013 31409573 PMC6796785

