# Supplementary figures and images for: Integrated bioinformatics analysis of nucleotide metabolism based molecular subtyping and biomarkers in lung adenocarcinoma
Source: Front Immunol. 2024 Aug 1;15:1430171. doi: 10.3389/fimmu.2024.1430171 (PMC11324481; doi:10.3389/fimmu.2024.1430171)

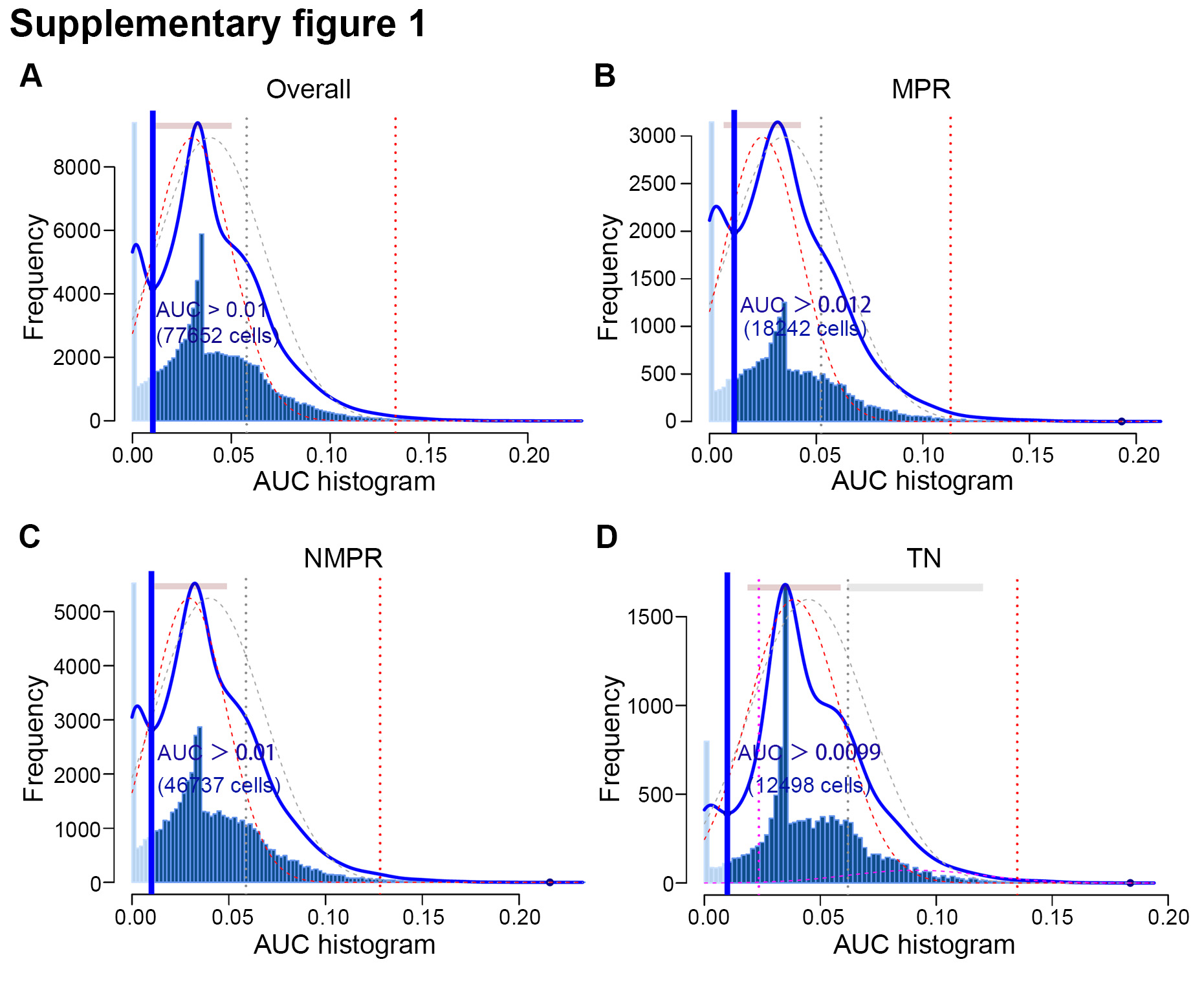

Supplement: Supplementary Figure 1 — (A–D) Thresholds for NMRS signature gene abundance across different groups set by the ‘AUCell’ algorithm. [file Image_1.jpeg]

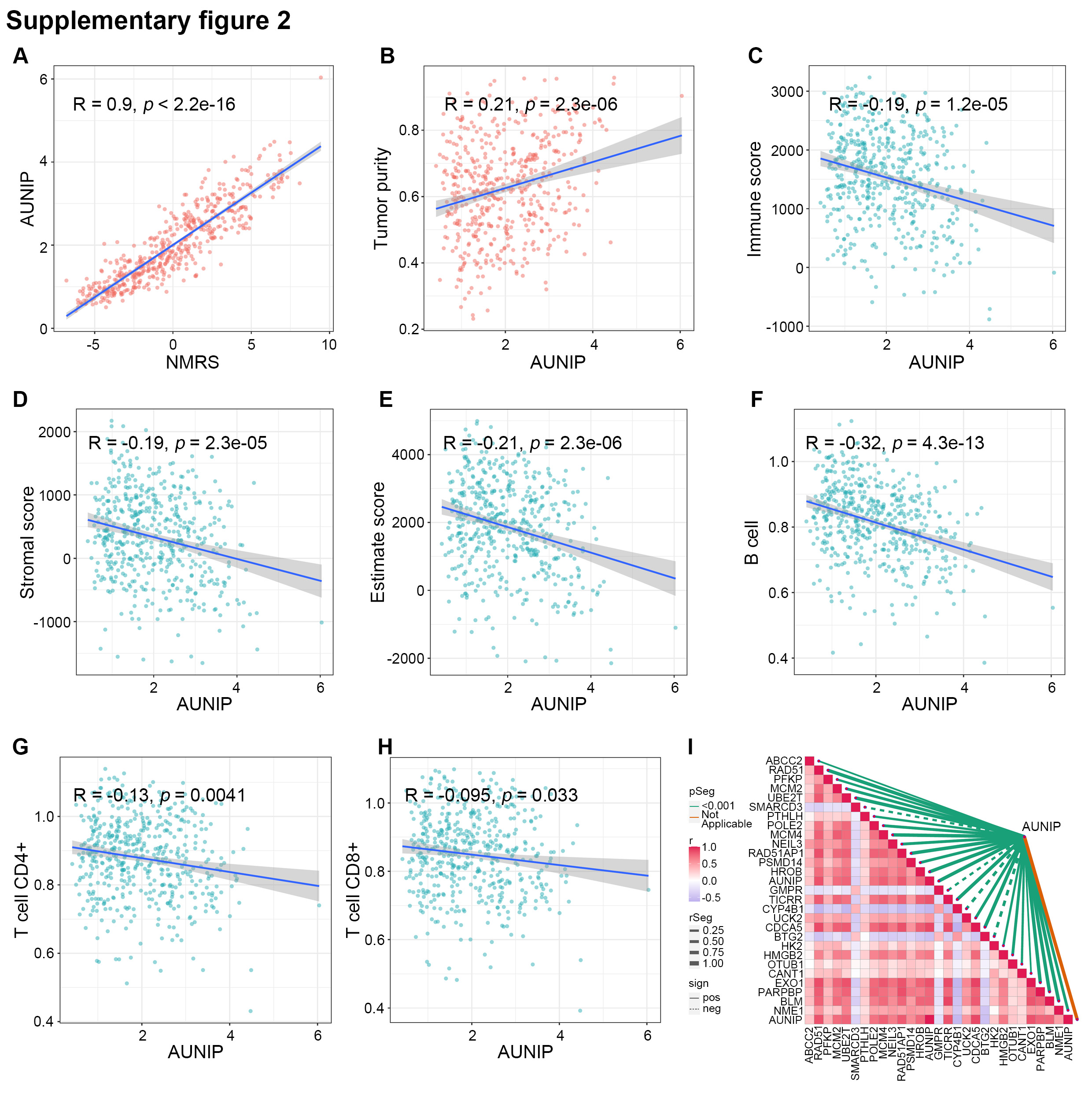

Supplement: Supplementary Figure 2 — (A) Correlation between AUNIP transcription levels and NMRS. (B–E): The ‘ESTIMATE’ algorithm revealed the correlation between AUNIP transcription levels and tumor purity (B), immune score (C), stromal score (D), and the overall ESTIMATE score (E). (F–H) Correlation between AUNIP and the relative abundance of three different immune cell types revealed by deconvolution algorithm. (I) Correlation of AUNIP transcription levels with the other 27 NMRS signature genes. [file Image_2.jpeg]

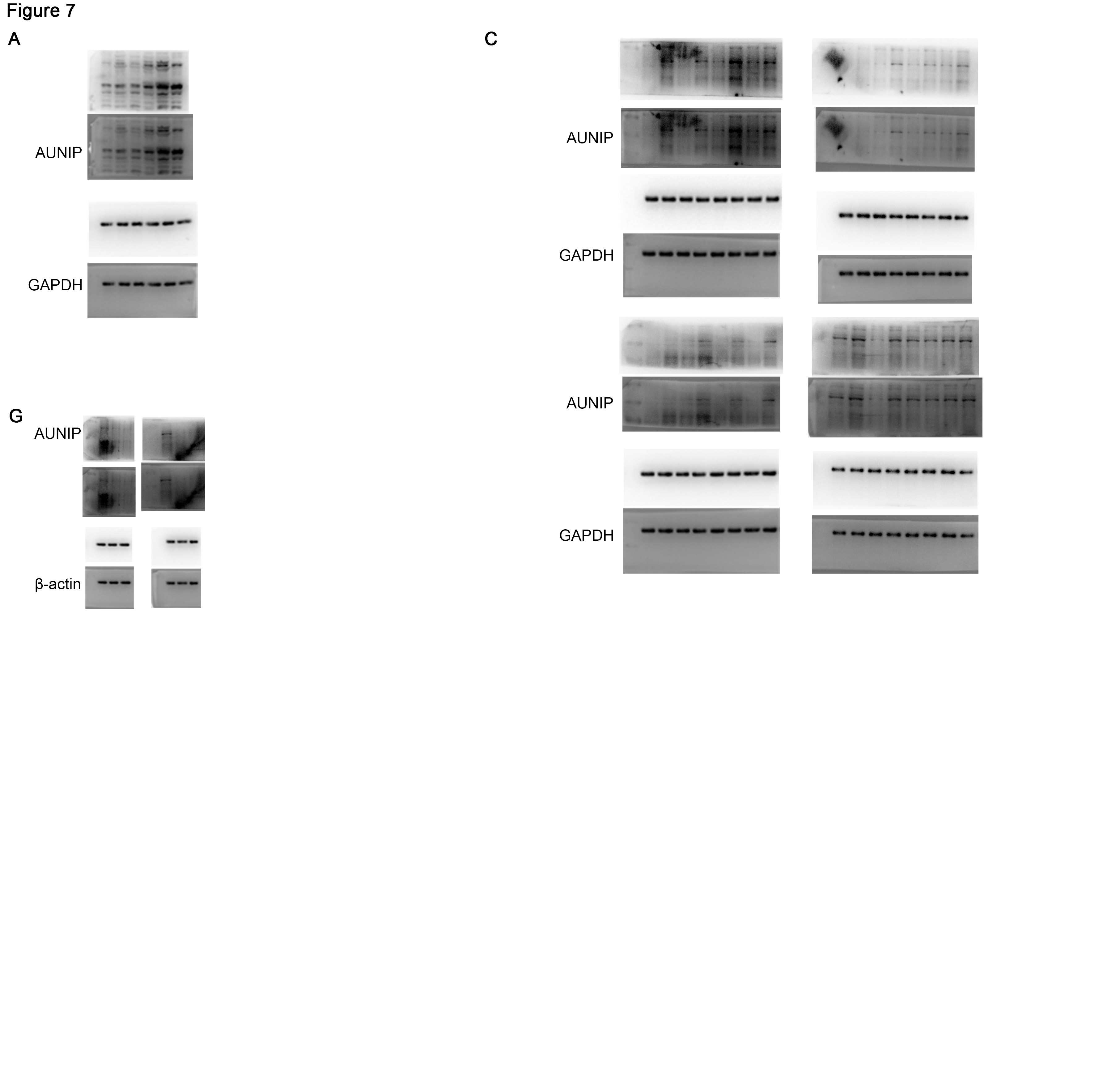

Supplement: Supplementary file 3 [file Image_3.jpeg]
